# Comparison of the use of prenatal care services and the risk of preterm birth between pregnant women with disabilities and those without disabilities: A nationwide cohort study

**DOI:** 10.3389/fpubh.2023.1090051

**Published:** 2023-01-27

**Authors:** Meng-Bin Tang, Pei-Tseng Kung, Li-Ting Chiu, Wen-Chen Tsai

**Affiliations:** ^1^Department of Public Health, Graduate Institute of Public Health, China Medical University, Taichung, Taiwan; ^2^Department of Health Services Administration, China Medical University, Taichung, Taiwan; ^3^Department of Family Medicine, China Medical University Hospital, Taichung, Taiwan; ^4^Department of Healthcare Administration, Asia University, Taichung, Taiwan; ^5^Department of Medical Research, China Medical University Hospital, China Medical University, Taichung, Taiwan

**Keywords:** prenatal care, preterm birth, pregnant women with disability, disabled women, disparity in prenatal care

## Abstract

**Objective:**

The difficulties faced by pregnant women with disabilities in accessing health care may make them less likely to receive prenatal care. The aims of this study were to compare the number of prenatal services and the risk of preterm birth between pregnant women with and without disabilities.

**Methods:**

A total of 2999 pregnant women aged ≥20 years with birth records in 2011–2014 in Taiwan were enrolled. Data were obtained from the Registration File for Physical and Mental Disabilities and the National Health Insurance Research Database. A 1:4 matching between pregnant women with disabilities and those without disabilities was performed. The logistic regression analysis with generalized estimating equations was used to analyze.

**Results:**

The median of prenatal care services used by pregnant women with disabilities was 9.00 (interquartile range, IQR: 2.00). Pregnant women with disabilities used fewer services than those without disabilities (median, 10.00; IQR: 1.00). The disabled group (8.44%) had a significantly higher proportion of preterm births than did the non-disabled group (5.40%). The disabled group was at a 1.30 times higher risk of preterm births than was the non-disabled group.

**Conclusions:**

Pregnant women with disabilities used significantly fewer prenatal care services and had a significantly higher risk of preterm birth than pregnant women without disabilities.

## Introduction

According to the Ministry of Health and Welfare, the number of people with physical and mental disabilities in Taiwan by the end of 2019 was 1.18 million, accounting for 5.03% of the population in Taiwan, and a yearly increasing trend (1). People with physical and mental disabilities are often disadvantaged in terms of healthcare and have multiple health problems and medical needs (2). Existing studies on prenatal care services and the risk of preterm birth among people with disabilities in Asian countries are few.

The Ministry of Health and Welfare of Taiwan provided 10 free prenatal care services for pregnant women Since 1995 (3). The prenatal care services included physician visits, prenatal testing, nutritional counseling, and sonography. The reported number of live births in 2019 by the Gender Equality Committee of the Executive Yuan was 172,567, and the average utilization rate of the 10 free prenatal care services was 94.3%, with 97.8% having undergone at least four prenatal care services (4). Some studies have analyzed the factors affecting the use of prenatal care services. A study on the use of prenatal care services among rural women suggested that the pregnant women who were younger, had a lower income, and had more than one child used few prenatal care services (5). The use of prenatal care services was lower among single mothers (6), those with lower incomes (7), those living in rural areas, and those with a lower level of education (8). A United States' study showed that 97% of women without hearing impairment used prenatal care services, while only 74% of women with hearing impairment used them (9). Recent studies indicated that women with intellectual/developmental disabilities and those with limited hearing delays initiating prenatal care, and the women with intellectual/developmental disabilities had the highest risk among different types of disabilities (10, 11).

According to the World Health Organization, preterm birth is defined as birth before 37 weeks of gestation (12). Over 15 million preterm births occur worldwide yearly, with a global percentage of 11% (13), India, China, Nigeria, Pakistan, Indonesia, and the United States account for half of the global preterm births in 2010 (14). Preterm birth is a major clinical issue that may cause fetal death, developmental delay, and physical and mental impairment in children (15). In Taiwan, the Health Promotion Administration survey in 2018 showed that 20,052 births occurred before 37 weeks of gestation (i.e., preterm birth), making up 10.94% of the total reported births (16). A study in China in 2018 showed that maternal age, history of miscarriage, and prenatal care were associated with preterm birth, and regular prenatal care was a protective factor in reducing preterm birth (17). Twelve European countries jointly published a study in 2015 in which the risk of preterm birth was 1.84 times higher among pregnant women with a low education level than in pregnant women with a higher education level (18). Studies conducted in other countries revealed that the risk of spontaneous miscarriage is higher in pregnant women with disabilities than in pregnant women without disabilities, and this risk increases with restrictions on mobility in this group (19). The percentage of preterm birth is also higher in pregnant women with disabilities than in pregnant women without disability (20). This study aimed to examine differences in the number of prenatal care services and the risk of preterm birth among women with and without disabilities in Taiwan.

## Materials and methods

### Data sources

Participant data were obtained from the Registry File for the Disabled People from the Ministry of the Interior, and the National Health Insurance Research Database (NHIRD) from the Ministry of Health and Welfare. In addition, to obtain participants' basic characteristics, health status, prenatal care history, and birth history (preterm birth and miscarriage), 2005–2010 data were obtained from the NHIRD.

### Research participants

This was retrospective cohort study. Pregnant women with disabilities and pregnant women without disabilities aged ≥20 years with birth records between 2011 and 2014 in Taiwan were eligible for the study; the birth records of 884,375 eligible women were analyzed during this period. The samples were not restricted to singleton and livebirth deliveries. There were many reasons for miscarriage, including spontaneous abortion and artificial abortion. Due to early termination of pregnancy, some women did not complete their prenatal care rendezvous and could not be considered as having a preterm birth. Therefore, women with miscarriages were excluded. In order to include one observation for each study participant during the study period, this study only selected women's first birth during the study period. A total of 2,999 pregnant women with disability and 630,024 pregnant women without disability were included in the analysis.

**Figure 1:**
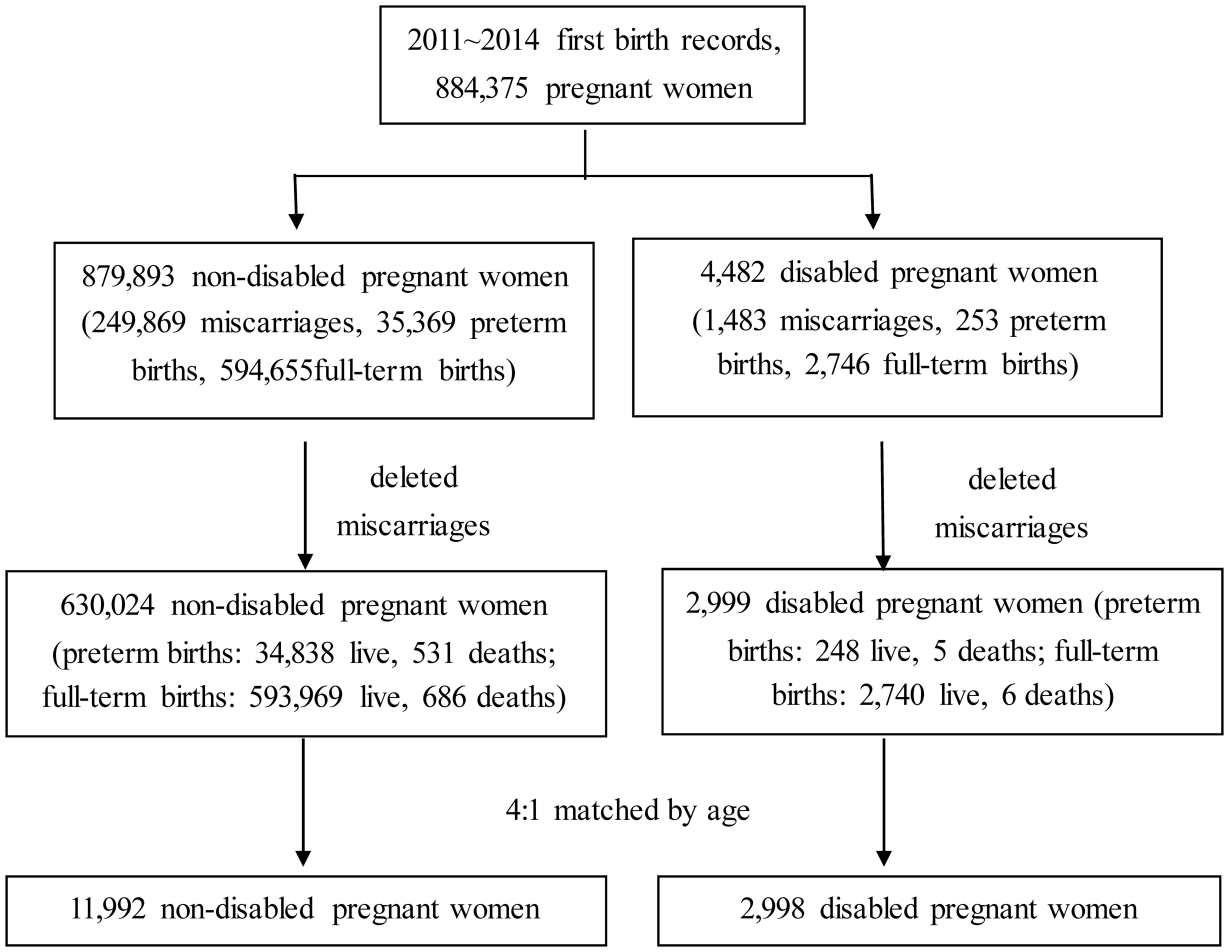
The sample collection process.

To reduce the age difference between the two groups, pregnant women with disabilities (hereafter the disabled group) and pregnant women without disabilities (hereafter the non-disabled group) were matched using the ratio of 1:4, with 2,998 and 11,992 participants in each group, respectively. The participant selection process is shown in Figure 1.

### Definition and description of variables

Physical and mental disabilities were divided into eight categories: moving functional limitation, visual impairment, hearing impairment, intellectual impairment, dysfunction of vital organs, multiple impairments, chronic mental illness, and other impairments (including voice or speech impairment, dementia, facial impairment, balance disorder, intractable epilepsy, autism, chromosomal abnormalities, congenital metabolic abnormalities, other congenital defects, and rare diseases). Dysfunction of vital organs included organs of heart, blood, respiratory organ, swallow function, stomach, intestine, liver, kidney, and urinary bladder. Multiple impairments were defined as people who had two or more impairments that were not for the same reason and were not related. We combined autism, chromosomal abnormalities, congenital metabolic abnormalities, other congenital defects, and rare diseases into the “other impairments” group because the total number of people was only 150. Impairment levels were classified as mild, moderate, severe, and profound. According to the assessment system of disability type and impairment level in Taiwan, two medical professionals are required to perform a detailed classification and assessment of the individuals' body function and structure, using the World Health Organization's International Classification of Functioning, Disability and Health. Non-physician health professionals evaluated the participants' activities, participation, and environment using the World Health Organization's International Classification of Functioning, Disability and Health (21, 22).

The baseline characteristics of pregnant women included age, monthly salary, education level, and degree of urbanization of their resident area. Monthly salary was divided into six levels: ≤ NT$17,280; NT$17,281–22,800; NT$22,801–28,800; NT$28,801–36,300; NT$36,301–45,800; and ≥NT$45,801. The classification by Liu et al. (23) was used to classify the degree of urbanization into seven levels, where level 1 was the most urbanized area and level 7 was the least urbanized area (23). Educational level was obtained from the Registry File for the Disabled People from the Ministry of the Interior and was classified into illiterate, elementary school, junior high school, high school (vocational), college, and university or higher.

The health status of the mothers was determined by the severity of comorbidities. The severity of comorbidities was determined by converting the primary and secondary International Classification of Diseases, 9th Revision, Clinical Modification (ICD-9)-CM codes of the study participants into numerical weight scores (score range, 0 to ≥2) using the Charlson Comorbidity Index (CCI) modified by Deyo et al.; the higher the score, the more severe the comorbidity (24).

Since 2005, preterm births and miscarriages have been monitored. The primary and secondary ICD-9-CM codes for preterm birth are 644.2x, 640.81, 640.91, and 641.21; while those for miscarriage are 632.xx, 634.xx-637.xx, and 656.41. Data on the use of prenatal care services were obtained during the free prenatal care services (IC41-IC50, IC51-IC60 in the Taiwan NHI database) for pregnant women covered by the National Health Insurance; these data were used to calculate the number of prenatal care services used and quantify their use during the current or past pregnancy.

### Statistical analysis

In this study, statistical analysis was performed using SAS 9.4 software (SAS Institute Inc., Cary, NC, USA), the numbers of prenatal care services and percentages of preterm birth among the disabled group and non-disabled group were expressed as numbers, percentage, means, standard deviation (SD), median, and interquartile range (IQR). The Wilcoxon rank-sum test, Wilcoxon signed rank test, and chi-square test were used to compare the differences in the number of prenatal care services and preterm births between the two groups. This study excluded women with preterm births when comparing the number of prenatal care services between the two groups.

The multivariate logistic regression analysis with generalized estimating equations (GEE) was used to compare the risk of preterm birth between two groups after controlling for age, monthly salary, degree of urbanization of the residence area, severity of comorbidities, previous use of prenatal care services, history of preterm birth, and history of miscarriage.

This study further performed the stratification analysis for the disabled group. Kruskal-Wallis test and Wilcoxon rank-sum test were used to compare the difference in the number of prenatal care services used among subgroups in pregnant women with disability. A multivariate logistic regression analysis was used to explore the factors associated with preterm birth in the disabled group. In this study, the occurrence or not of preterm birth was used as a dependent variable, while the type of impairment, impairment level, age, monthly salary, education level, degree of urbanization of the residence area, severity of comorbidities, use of prenatal care services during pregnancy, history of preterm birth, and history of miscarriage were used as independent variables.

This study was reviewed and approved by the Research Ethics Center of authors' affiliated organization (IRB No. CMUH105-REC2-020).

## Results

To reduce the age difference between the two groups, a 4:1 matching of 11,992 pregnant women without disabilities to 2,998 pregnant women with disabilities was performed (Table 1). The median of prenatal care services was 10.00 (IQR 1.00) and 9.00 (IQR 2.00), respectively (Table 2), and the average number of prenatal care services in the two groups became closer after excluding women with preterm births (9.05 ± 1.97 vs. 8.52 ± 2.62). The disabled group used fewer prenatal care services than the non-disabled group, when comparing different factors (*P* < 0.05): the number of services used was significantly lower in older women, women with a lower monthly salary, more severe comorbidities, history of preterm birth, and living in an area with a lower degree of urbanization (Table 2). Notably, the number of prenatal care services used was lower in those with a higher maternal age.

**Table 1:** Age distribution before and after pairing of disabled pregnant women and non-disabled pregnant women.

		**Before**					**After**				
		**Non-disabled**		**Disabled**			**Non-disabled**		**Disabled**		
		***n***	**%**	***n***	**%**	***p*** **-value** ^a^	***n***	**%**	***n***	**%**	***p*** **-value** ^a^
Sum		630,024	99.53	2,999	0.47		11,992	80.00	2,998	20.00	
**Age (years)**						< 0.001					1.000
	< 30	207,388	99.45	1,149	0.55		4,592	80.00	1,148	20.00	
	30–34	271,313	99.59	1,126	0.41		4,504	80.00	1,126	20.00	
	35–39	127,864	99.56	570	0.44		2,280	80.00	570	20.00	
	≧40	23,459	99.35	154	0.65		616	80.00	154	20.00	

a Chi-Squared test.

**Table 2:** Comparison of the utilization of prenatal care services between disabled pregnant women and non-disabled pregnant women (excluding those with preterm births).

		**Non-disabled women**					**Disabled women**					
**Variable**		***N***	**Mean**	**SD**	**Median**	**IQR**	***N***	**Mean**	**SD**	**Median**	**IQR**	***p*** **-value** ^a^
Total		10,980	9.05	1.97	10.00	1.00	2,745	8.52	2.62	9.00	2.00	< 0.001^b^
**Age**												
	< 30 y/o	4,220	9.07	1.96	10.00	1.00	1,055	8.48	2.61	10.00	3.00	< 0.001
	30–34 y/o	4,140	9.17	1.79	10.00	1.00	1,035	8.79	2.32	10.00	2.00	< 0.001
	35–39 y/o	2,072	9.02	1.88	10.00	1.00	518	8.43	2.64	9.00	2.00	< 0.001
	≧40 y/o	548	8.09	3.14	9.00	3.00	137	7.04	3.98	9.00	5.00	0.007
**Monthly salary (NTD)**												
	≦17,280	1,127	8.18	2.78	9.00	2.00	742	7.39	3.26	8.00	5.00	< 0.001
	17,281–22,800	4,423	9.07	1.92	10.00	1.00	1,148	8.92	2.23	10.00	2.00	0.001
	22,801–28,800	1,221	9.26	1.82	10.00	1.00	279	9.10	1.89	10.00	1.00	0.119
	28,801–36,300	1,448	9.31	1.55	10.00	1.00	263	8.87	2.25	10.00	2.00	0.003
	36,301–45,800	1,234	9.23	1.76	10.00	1.00	165	8.88	2.27	10.00	2.00	0.010
	>45,800	1,527	9.11	1.88	10.00	1.00	148	8.90	2.41	10.00	2.00	0.152
**Degree of urbanization of residence**												
	1	3,429	9.06	1.97	10.00	1.00	643	8.60	2.63	10.00	2.00	< 0.001
	2	3,753	9.12	1.88	10.00	1.00	806	8.82	2.43	10.00	2.00	< 0.001
	3	2,035	9.02	2.04	10.00	1.00	531	8.52	2.56	9.00	2.00	< 0.001
	4	1,173	9.05	1.95	10.00	1.00	423	8.52	2.54	9.00	2.00	< 0.001
	5	98	8.96	2.20	10.00	2.00	59	7.76	3.29	9.00	4.00	0.005
	6	212	8.50	2.44	9.00	2.00	141	7.60	2.94	8.00	4.00	< 0.001
	7	280	8.75	2.18	10.00	1.00	142	7.61	3.04	9.00	4.00	< 0.001
**CCI**												
	0	1,0277	9.06	1.97	10.00	1.00	2,432	8.56	2.57	10.00	2.00	< 0.001
	1	628	9.05	1.93	10.00	2.00	239	8.21	3.00	9.00	3.00	< 0.001
	≧2	75	8.68	3.05	9.00	2.00	74	7.95	3.10	8.00	4.50	0.003
**Previous experience with prenatal care**												
	No	7,355	9.07	2.03	10.00	1.00	1,773	8.57	2.62	10.00	2.00	< 0.001
	Yes	3,625	9.02	1.84	10.00	1.00	972	8.42	2.64	9.00	2.00	< 0.001
**Experience of preterm birth**												
	No	10,828	9.05	1.98	10.00	1.00	2,678	8.57	2.56	10.00	2.00	< 0.001
	Yes	152	8.97	1.75	9.00	3.00	67	6.48	3.94	7.00	5.00	0.001
**Experience of miscarriage**												
	No	9,372	9.04	2.00	10.00	1.00	2,327	8.49	2.69	10.00	2.00	< 0.001
	Yes	1,608	9.14	1.78	10.00	1.00	418	8.67	2.24	9.00	2.00	< 0.001

a Wilcoxon rank-sum test for the median of prenatal care used between disabled pregnant women and non-disabled pregnant women.

b Wilcoxon signed rank test for the median of prenatal care used between two matched groups.

N, number; SD, standard deviation; y/o, years old; NTD, New Taiwan dollar; CCI, Charlson comorbidity index.

The differences in the proportion of preterm births for each variable between the two groups are shown in Table 3. The proportion of preterm birth was significantly higher in the disabled group (8.44%) than in the non-disabled group (5.40%). The proportion was higher in the disabled group than in the non-disabled group in all age groups. The proportion of preterm birth was higher in the disabled group than in the non-disabled group under different monthly salary conditions, but the proportion in the disabled group differed from that in the non-disabled group. The proportion of preterm birth in the disabled group was higher than that in the non-disabled group in terms of the degree of urbanization of the residence areas, except for lower degree of urbanization of the residence areas (Levels 5–7). The proportion of preterm birth in the disabled group was higher than that in the non-disabled group under the same severity of comorbidities (*p* < 0.05). Regardless of a past history of use of prenatal care services or miscarriage history, the proportion of women without a history of use of prenatal care services was higher than in those with one (14.93 vs. 9.78%), and the proportion among those with a history of miscarriage was higher than among those without one (11.06 vs. 5.46%). However, the proportion of preterm birth had no significant difference between the non-disabled group (20.21%) and the disabled group (22.09%) with a preterm birth history.

**Table 3:** Comparison of the risk of preterm birth between disabled pregnant women and non-disabled pregnant women.

		**Non-disabled women**		**Disabled women**						
		**Preterm birth**		**Preterm birth**						
**Variable**		***n***	**%**	***n***	**%**	***p*** **-value** ^a^	**aOR**	**95% CI**		***p*** **-value**
Sum		647	5.40	253	8.44	< 0.001				
**Identity**										
	Non-disabled women						1.00	-	-	-
	Disabled women						1.30	1.10	1.55	0.003
**Age**										
	< 30 y/o	216	4.70	93	8.10	< 0.001	1.00	-	-	-
	30–34 y/o	235	5.22	91	8.08	< 0.001	1.08	0.91	1.27	0.371
	35–39 y/o	157	6.89	52	9.12	0.081	1.32	1.09	1.60	0.004
	≧40 y/o	39	6.33	17	11.04	0.066	1.17	0.86	1.59	0.322
**Monthly salary (NTD)**										
	≦17,280	81	6.44	93	11.14	< 0.001	1.00	-	-	-
	17,281–22,800	251	5.13	85	6.89	0.018	0.73	0.60	0.89	0.002
	22,801–28,800	67	5.20	23	7.62	0.135	0.79	0.60	1.04	0.086
	28,801–36,300	75	4.97	23	8.04	0.051	0.73	0.56	0.96	0.023
	36,301–45,800	68	5.08	15	8.33	0.104	0.74	0.56	0.98	0.038
	>45,800	105	6.16	14	8.64	0.285	0.83	0.64	1.08	0.164
**Urbanization of residence**										
	1	190	5.17	53	7.61	0.013	1.00	-	-	-
	2	242	5.85	79	8.93	0.001	1.16	0.98	1.38	0.092
	3	104	4.73	50	8.61	< 0.001	0.98	0.79	1.21	0.853
	4	71	5.44	38	8.24	0.042	1.07	0.85	1.36	0.563
	5	3	2.65	6	9.23	0.075	0.81	0.41	1.62	0.551
	6	20	7.46	15	9.62	0.553	1.26	0.86	1.85	0.238
	7	17	5.76	12	7.79	0.530	1.03	0.68	1.54	0.904
**CCI**										
	0	589	5.25	194	7.39	< 0.001	1.00	-	-	-
	1	50	7.36	37	13.41	0.005	1.51	1.19	1.91	0.001
	≧2	8	8.60	22	22.92	0.013	1.99	1.29	3.07	0.002
**Prenatal care this pregnancy**										
	No	22	9.78	20	14.93	0.194	1.00	-	-	-
	Yes	625	5.31	233	8.14	< 0.001	0.60	0.43	0.85	0.004
**Experience of preterm birth**										
	No	609	5.16	234	8.04	< 0.001	1.00	-	-	-
	Yes	38	20.21	19	22.09	0.845	3.74	2.75	5.08	< 0.001
**Experience of miscarriage**										
	No	556	5.39	201	7.95	< 0.001	1.00	-	-	-
	Yes	91	5.46	52	11.06	< 0.001	1.13	0.94	1.36	0.205

a Chi-Squared test.

N, number; OR, adjusted odds ratio; CI, confidence interval; y/o, years old; NTD, New Taiwan dollar.

The relative risk of preterm births was further compared between the two groups using a logistic regression model to control for related factors (Table 3). After controlling for related factors, the risk of preterm birth in the disabled group was 1.30 (95% confidence interval [CI]: 1.10–1.55) times higher than that in the non-disabled group.

This study further conducted the stratification analysis for the disabled group. There were 2,999 women in the disabled group, and 95.53% of this group used prenatal care services (median 9.00, IQR 2.00; mean 8.30 ± 2.74). When analyzing the use of prenatal care services in the disabled group (Table 4), women with hearing impairment used the highest number of prenatal care services (median 10.00, IQR 1.00; mean 9.02 ± 2.09), followed by women with other impairments (median 10.00, IQR 1.00; mean 8.88 ± 2.20) and visual impairment (median 10.00, IQR 1.00; mean 8.71 ± 2.45). Women with intellectual impairment (median 9.00, IQR 4.00; mean 7.46 ± 3.17) used the least number of services, followed by women with a chronic mental illness (median 9.00, IQR 3.00; mean 8.00 ± 2.99). In the disabled group, women aged 30–34 years (median 10.00, IQR 2.00; mean 8.57 ± 2.46) used the prenatal care services the most, while those aged ≥40 years (median 9.00, IQR 5.00; mean 6.94 ± 3.87) used them the least. Women with a lower income or more severe comorbidities used the services less. Meanwhile, women with a higher education level or those living in more urbanized areas used the services more. Women who had a preterm birth (median 7.00, IQR 5.00; mean 6.36 ± 3.82) used services less than those without (median 10.00, IQR 2.00; mean 8.35 ± 2.68). Prenatal and miscarriage histories did not affect the average number of prenatal care services used by the disabled group (*p* > 0.05).

**Table 4:** Number of prenatal care services used by pregnant women with disability.

**Variable**		**N**	**Mean**	**SD**	**Median**	**IQR**	***p*-value^b^**
Total		2,999	8.30	2.74	9.00	2.00	-
**Type of impairment**							< 0.001
	Moving functional limitation	1,094	8.60	2.49	10.00	2.00	
	Visual impairment	134	8.71	2.45	10.00	1.00	
	Hearing impairment	306	9.02	2.09	10.00	1.00	
	Intellectual impairment	708	7.46	3.17	9.00	4.00	
	Multiple impairments	171	8.32	2.81	9.00	2.00	
	Dysfunction of vital organs	188	8.12	2.67	9.00	3.00	
	Chronic mental illness	248	8.00	2.99	9.00	3.00	
	Other impairments^a^	150	8.88	2.20	10.00	1.00	
**Level of impairment**							< 0.001
	Mild	1,724	8.43	2.58	10.00	2.00	
	Moderate	871	8.02	2.98	9.00	3.00	
	Severe	312	8.38	2.73	10.00	2.00	
	Profound	92	8.08	2.99	9.00	3.00	
**Age**							< 0.001
	< 30 y/o	1,149	8.24	2.77	10.00	3.00	
	30–34 y/o	1,126	8.57	2.46	10.00	2.00	
	35–39 y/o	570	8.22	2.70	9.00	2.00	
	≧40 y/o	154	6.94	3.87	9.00	5.00	
**Monthly salary (NTD)**							< 0.001
	≦17,280	846	7.12	3.33	8.00	5.00	
	17,281–22,800	1,242	8.75	2.32	10.00	2.00	
	22,801–28,800	290	8.95	2.00	10.00	1.00	
	28,801–36,300	285	8.71	2.32	10.00	2.00	
	36,301–45,800	172	8.73	2.37	10.00	2.00	
	>45,800	164	8.60	2.62	10.00	2.00	
**Degree of urbanization of residence**							< 0.001
	1	696	8.40	2.71	10.00	2.00	
	2	885	8.56	2.60	10.00	2.00	
	3	581	8.29	2.71	9.00	2.00	
	4	461	8.33	2.65	9.00	2.00	
	5	65	7.57	3.32	9.00	4.00	
	6	156	7.32	3.01	8.00	4.00	
	7	155	7.49	3.02	9.00	4.00	
**Education level**							< 0.001
	Illiterate and elementary school	546	7.98	3.02	9.00	3.00	
	Junior high school	609	8.12	2.87	9.00	3.00	
	High school (vocational)	893	8.33	2.69	9.00	2.00	
	College and university or higher	483	8.84	2.14	10.00	1.00	
	Unclear	468	8.27	2.77	9.00	2.00	
**CCI**							< 0.001
	0	2,614	8.37	2.68	10.00	2.00	
	1	286	7.95	3.05	9.00	3.00	
	≧2	99	7.40	3.10	8.00	4.50	
**Previous experience with prenatal care**							0.143
	No	1,958	8.31	2.75	10.00	2.00	
	Yes	1,041	8.27	2.71	9.00	2.00	
**Experience of preterm birth**							< 0.001
	No	2,913	8.35	2.68	10.00	2.00	
	Yes	86	6.36	3.82	7.00	5.00	
**Experience of miscarriage**							0.378
	No	2,529	8.28	2.79	10.00	2.00	
	Yes	470	8.40	2.42	9.00	2.00	

a Including voice or speech impairment, dementia, facial impairment, balance disorder, intractable epilepsy, autism, chromosomal abnormalities, congenital metabolic abnormalities, other congenital defects, and rare diseases.

b Comparing the median of prenatal care used using Kruskal-Wallis test for more than two groups and using Wilcoxon rank-sum test for two groups.

N, number; SD, standard deviation; y/o, years old; NTD, New Taiwan dollar; CCI, Charlson comorbidity index.

The distribution of preterm births among the variables and the related risk factors of preterm birth in the disabled group are shown in Table 5. Approximately 8.44% of women in the disabled group had preterm births. Regarding preterm births and related factors, 21.28% of women with vital organ dysfunction, 6.00% of those with other impairments (the lowest), and 7.04% of those with moving functional limitation had preterm births. The risk of preterm birth was significantly higher among women with vital organ dysfunction than those with moving functional limitation (adjusted odds ratio [aOR] = 2.58, 95% CI: 1.59–4.18). The impairment level was not directly proportional to the percentage of preterm birth; however, 16.30% of women with profound impairment and 7.54% of those with mild impairment had preterm births; the difference was significant. The proportion of preterm births was higher among older women. Women with the lowest monthly salary (≤ NT$17,280) had the highest proportion of preterm birth (11.23%). There was no significant difference in the proportion of preterm birth in terms of education level and degree of urbanization of the area of residence. The proportion of preterm births among women with more severe comorbidities (CCI ≥ 2) was significantly higher than those with less severe comorbidities (CCI = 0; 23.23 vs. 7.35%). The proportion of preterm births in women who did not use prenatal care services during pregnancy was 14.93%. The proportion of preterm births in women with a preterm birth history was 22.09%, which was 2.68 times greater than in those without a preterm birth history (aOR = 2.68, 95% CI: 1.52–4.70). The proportion among those with a history of miscarriage was 11.06%, which was significantly higher than that among women without a history of miscarriage (aOR = 1.49, 95% CI: 1.07–2.08).

**Table 5:** The risk of preterm birth and related factors among pregnant women with disability.

		**Full-term birth**		**Preterm birth**						
**Variable**		***n***	**%**	***n***	**%**	***p*** **-value** ^b^	**aOR**	**95% CI**		***p*** **-value**
Sum		2,746	91.56	253	8.44	-				
**Type of impairment**						< 0.001				
	Moving functional limitation	1,017	92.96	77	7.04		1.00	-	-	-
	Visual impairment	124	92.54	10	7.46		1.20	0.59	2.44	0.921
	Hearing impairment	281	91.83	25	8.17		1.43	0.87	2.37	0.343
	Intellectual impairment	651	91.95	57	8.05		1.14	0.76	1.72	0.927
	Multiple impairments	156	91.23	15	8.77		1.07	0.54	2.15	0.783
	Dysfunction of vital organs	148	78.72	40	21.28		2.58	1.59	4.18	< 0.001
	Chronic mental illness	228	91.94	20	8.06		0.75	0.41	1.39	0.101
	Other impairments^a^	141	94.00	9	6.00		0.82	0.39	1.71	0.270
**Level of impairment**						0.018				
	Mild	1,594	92.46	130	7.54		1.00	-	-	-
	Moderate	790	90.70	81	9.30		1.10	0.80	1.49	0.914
	Severe	285	91.35	27	8.65		0.93	0.57	1.52	0.332
	Profound	77	83.70	15	16.30		1.51	0.70	3.26	0.279
**Age**						0.561				
	< 30 y/o	1,056	91.91	93	8.09		1.00	-	-	-
	30–34 y/o	1,035	91.92	91	8.08		1.07	0.77	1.49	0.944
	35–39 y/o	518	90.88	52	9.12		1.09	0.73	1.62	0.954
	≧40 y/o	137	88.96	17	11.04		1.17	0.63	2.15	0.726
**Monthly salary (NTD)**						0.016				
	≦17,280	751	88.77	95	11.23		1.00	-	-	-
	17,281–22,800	1,159	93.32	83	6.68		0.60	0.43	0.83	0.130
	22,801–28,800	268	92.41	22	7.59		0.67	0.40	1.13	0.663
	28,801–36,300	260	91.23	25	8.77		0.79	0.48	1.31	0.698
	36,301–45,800	157	91.28	15	8.72		0.76	0.40	1.44	0.868
	>45,800	151	92.07	13	7.93		0.64	0.33	1.25	0.611
**Degree of urbanization of residence**						0.968				
	1	643	92.39	53	7.61		1.00	-	-	-
	2	806	91.07	79	8.93		1.22	0.84	1.78	0.483
	3	531	91.39	50	8.61		1.15	0.76	1.76	0.791
	4	423	91.76	38	8.24		1.11	0.70	1.75	0.987
	5	59	90.77	6	9.23		1.20	0.48	3.00	0.836
	6	141	90.38	15	9.62		1.14	0.60	2.17	0.911
	7	143	92.26	12	7.74		0.94	0.48	1.87	0.569
**Education level**						0.804				
	Illiterate and elementary school	505	92.49	41	7.51		1.00	-	-	-
	Junior high school	553	90.80	56	9.20		1.16	0.75	1.80	0.645
	High school (vocational)	813	91.04	80	8.96		1.25	0.83	1.90	0.246
	College and university or higher	444	91.93	39	8.07		1.04	0.62	1.73	0.766
	Unclear	431	92.09	37	7.91		1.03	0.63	1.66	0.685
**CCI**						< 0.001				
	0	2,422	92.65	192	7.35		1.00	-	-	-
	1	248	86.71	38	13.29		1.72	1.17	2.53	0.498
	≧2	76	76.77	23	23.23		2.17	1.23	3.84	0.087
**Prenatal care this pregnancy**						0.010				
	No	114	85.07	20	14.93		1.00	-	-	-
	Yes	2,632	91.87	233	8.13		0.65	0.38	1.11	0.114
**Experience of preterm birth**						< 0.001				
	No	2,679	91.97	234	8.03		1.00	-	-	-
	Yes	67	77.91	19	22.09		2.68	1.52	4.70	0.001
**Experience of miscarriage**						0.030				
	No	2,328	92.05	201	7.95		1.00	-	-	-
	Yes	418	88.94	52	11.06		1.49	1.07	2.08	0.020

a Including voice or speech impairment, dementia, facial impairment, balance disorder, intractable epilepsy, autism, chromosomal abnormalities, congenital metabolic abnormalities, other congenital defects, and rare diseases.

b Chi-Squared test.

N, number; aOR, adjusted odds ratio; CI, confidence interval; y/o, years old; NTD, New Taiwan dollar; CCI, Charlson comorbidity index.

## Discussion

A median of 10.00 (IQR 1.00) prenatal care services were used by pregnant women without disabilities, while pregnant women with disabilities used a median of 9.00 (IQR 2.00) prenatal care services (*p* < 0.05); the difference was significant. Taiwan's National Health Insurance provided 10 free prenatal care services, although pregnant women could receive more than 10 prenatal care services and pay partially for the extra services. This study may provide the government an opportunity to increase the use of prenatal care services among pregnant women with disabilities. The average number of prenatal care services used by the disabled group was lower than that used by the non-disabled group; the number of prenatal care services used by the former was lower than that used by the latter in terms of age, monthly salary, degree of urbanization of the area of residence, severity of comorbidities, prior use of prenatal care services, history of preterm birth, and history of miscarriage. There was a health care disparity for the disabled group in terms of the use of prenatal care, but it was not very great under the universal health insurance system in Taiwan. This remains a concern for the government and social welfare units. Pregnant women with disabilities require counseling to increase their use of prenatal care services. Older pregnant women without disabilities used prenatal care services less frequently, which is also an issue requiring active counseling. The high risk of obstetric complications and lower use of prenatal care services among older women, when compared to younger women, may increase the incidence of adverse outcomes for both the mother and the fetus (25).

The utilization of prenatal care services by pregnant women with disabilities has been studied per previous literature. The results of this study on the utilization rate of prenatal care services were similar to those of a Korean study (26), which reported that pregnant women with disabilities used prenatal care services significantly lower than pregnant women without disabilities. However, this finding was contrary to that in a British study wherein the number of prenatal care services used by pregnant women with disabilities was significantly higher than that used by pregnant women without disabilities (27). The number of prenatal care services used by pregnant women with disabilities can be higher than that used by pregnant women without disabilities due to the differences in the cultural environments and social resources, as well as the different levels of accessible space and auxiliary resources of the pregnant women with disabilities. Therefore, the use of prenatal care services by pregnant women with disabilities in Taiwan needs to be improved. In addition to the improvement of the hardware environment for this group, social welfare interventions by social workers and volunteers can improve the use of prenatal care services and reduce the risk of preterm birth (28).

A comparison of the risk of preterm birth between pregnant women with and without disabilities revealed that the risk in the former was 1.30 times higher than that in the latter. Therefore, pregnant women with disabilities have a higher risk of preterm births and are a group to be concerned about. Several studies on preterm births in pregnant women with disabilities revealed similar results; the risk of preterm birth in pregnant women with disabilities was higher than that in pregnant women without disabilities (29–31). No studies have demonstrated a direct correlation between prenatal care and preterm birth until now.

This study examined the use of prenatal care services among pregnant women with different types of impairments, and women with intellectual impairment used the services the least (median 9.00, IQR 4.00). This may be due to multiple factors, including unknown information about prenatal care, insufficient social or structural factors (i.e., lack of sexual health education) and family support (32). In this study, women with hearing impairment used the prenatal care services highest; this finding differed from that reported in a United States' study, wherein the use of prenatal care services among women with hearing impairment was relatively low (10). This indicates that the Taiwanese environment may be more friendly and supportive of women with hearing impairment, compared to other impairment groups. The low use of prenatal care services among women with chronic mental illness is second only to the use among mothers with intellectual impairment; it is hypothesized that the loss of autonomy and expertise due to lesions in the brain in both groups makes it more difficult to receive prenatal care (33–35). This study found that the level of impairment was not inversely related to the use of prenatal care services, suggesting that the type of impairment has a greater effect than the level of impairment.

In the present study, older women in the disabled group, especially those above 40 years old, used the prenatal care services least (median 9.00, IQR 5.00; mean 6.94 ± 3.87). This is a noteworthy phenomenon, as older mothers have a higher risk of obstetric diseases, and pregnant women with disabilities should use prenatal care services (25). Our study results showed that in the disabled group, women with a higher salary, higher educational level, or women living in areas with a higher degree of urbanization used the services more; therefore, use of prenatal services may be related to better personal knowledge and social resources (5, 7, 8). A previous experience of use of prenatal care services and history of miscarriage did not affect the number of prenatal care services used by pregnant women with disabilities. The reasons for this are unclear, but a history of preterm birth was significantly associated with a lower use of prenatal care services, which suggests that pregnant women with disabilities and a preterm birth history should receive more active assistance during prenatal care. Most women with disabilities (95.53%) did access some level of prenatal care, but the study found some gaps in accessing care for women with disabilities.

The analysis of preterm births and related factors in the disabled group revealed that the risk of preterm birth was different in women with different impairment types. The highest proportion of preterm births was reported among women with a vital organ dysfunction, which increases the vulnerability to having a preterm birth. Pregnant women with organ dysfunction could increase the risk to the fetus (36, 37). We strongly recommend that obstetricians require special attention to the risk of preterm birth among women with vital organ dysfunction. Physical and mental impairment levels did not significantly affect the proportion of preterm births, which suggests that the impairment type has a greater effect than the level of impairment. Age, monthly salary, education level, and degree of urbanization of the area of residence did not significantly affect the proportion of preterm birth, reflected by the variation in the number of prenatal care services used. In the disabled group, women with more severe comorbidities had higher proportion of preterm birth than those with less severe comorbidities, which may be due to the poorer health condition of these women (38, 39). Although a history of miscarriage increased the number of prenatal care services used during the current pregnancy, a history of miscarriage was associated with preterm birth.

### Strengths and limitations

This study had some strengths. First, a national sample of women over a 4-year period (2011–2014) was used. Second, 17 types of impairment and four levels of impairment were included. Third, the study considered the effect of previous birth experiences, including factors such as previous use of prenatal care services, history of preterm birth, and history of miscarriage. Fourth, the study compared the number of prenatal care services and the risk of preterm birth under each variable using a ratio of 1:4 by age between pregnant women with disabilities and pregnant women without disabilities.

This study had some limitations. First, it had a retrospective design and used secondary data. Administrative health data does not capture the quality of care received, only the quantity. Second, this study included women covered by the National Health Insurance in Taiwan. The health insurance system in other countries may not be exactly the same as that in Taiwan; thus, the results may not be generalizable to other countries. Third, this study investigated the number of prenatal care services used, but we did not include the timing of prenatal care visits. Fourth, miscarriages were not included in the analysis. Finally, the twin pregnancy/delivery was rare, and we did not list it as a covariate in the analysis.

## Conclusions

The utilization of prenatal care services in pregnant women with disabilities was significantly lower than in pregnant women without disabilities. Women with intellectual impairment used the least number of prenatal services among all types of impairments. There was significantly higher risk of preterm birth among pregnant women with disabilities than those without disabilities. Factors such as type of impairment, history of preterm birth, and history of miscarriage were also associated with the risk of preterm birth in pregnant women with disabilities. These deserve more attention from the appropriate government agencies, and the results of this study can be used as a reference when formulating policies on prenatal care for pregnant women with disabilities.

## Data availability statement

Publicly available datasets were analyzed in this study. This data can be found here: The data that support the findings of this study are available from the National Health Insurance Research Database published by the Ministry of Health and Welfare (https://www.mohw.gov.tw/np-108-2.html), Taiwan, but restrictions apply to the availability of these data, which were used under license for the current study, and so are not publicly available. Due to legal restrictions imposed by the Taiwanese government related to the Personal Information Protection Act, the database cannot be made publicly available. Any raw data are not allowed to be removed from the Health and Welfare Data Science Center. The restrictions prohibited the authors from making the minimal data set publicly available. Data are however available from the authors upon reasonable request and with permission of the Health and Welfare Data Science Center, the Ministry of Health and Welfare, Taiwan.

## Ethics statement

The studies involving human participants were reviewed and approved by the Research Ethics Center of China Medical University Hospital (IRB No. CMUH105-REC2-020). Written informed consent for participation was not required for this study in accordance with the national legislation and the institutional requirements.

## Author contributions

M-BT, W-CT, L-TC, and P-TK conceived and designed the study and contributed data analysis and interpretation. M-BT, W-CT, and L-TC collected data. W-CT and P-TK obtained the funding source. M-BT, W-CT, and P-TK drafted the article and revised the article. All authors read and approved the final manuscript.

## References

[1] Number of people with physical and mental disabilities quarterly. Department of Statistics, Ministry of Health and Welfare. (2021). Available online at: https://dep.mohw.gov.tw/dos/cp-5224-62359-113.html (accessed March 9, 2021).

[2] Beange H McElduff A Baker W. Medical disorders of adults with mental retardation: a population study. Am J Ment Retard. (1995) 99:595–604. 7632427

[3] Prenatal care items and subsidies for pregnant women. Health Promotion Administration, Ministry of Health and Welfare. (2018). Available online at: https://www.hpa.gov.tw/Pages/Detail.aspx?nodeid=1143&pid=6596&sid=6598 (accessed October 9, 2018).

[4] The utilization of prenatal care services. Gender Equality Committee of the Executive Yuan. (2021). Available online at: https://www.gender.ey.gov.tw/gecdb/Stat_Statistics_DetailData.aspx?sn=iGZNg9VGCQqgVUqoT%24iZ7w%40%40 (accessed January 5, 2021).

[5] Nwaru BI Wu Z Hemminki E. Determinants of the use of prenatal care in rural China: the role of care content. Matern Child Health J. (2012) 16:235–41. 10.1007/s10995-010-0734-0 21184157

[6] Alves E Silva S Martins S Barros H. Family structure and use of prenatal care. Cad Saúde Pública, Rio de Janeiro. (2015) 31:1298–304. 10.1590/0102-311X00052114 26200376

[7] Schillaci MA Waitzkin H Carson EA Romain SJ. Prenatal care utilization for mothers from low-income areas of New Mexico, 1989–1999. PLoS ONE. (2010) 5:e12809. 10.1371/journal.pone.0012809 20862298 PMC2941446

[8] Alexandre PK Saint-Jean G Crandall L Fevrin E. Prenatal care utilization in rural areas and urban areas of Haiti. Rev Panam Salud Publica. (2005) 18:84–92. 10.1590/s1020-49892005000700002 16156958

[9] O'Hearn A. Deaf women's experiences and satisfaction with prenatal care: a comparative study. Fam Med. (2006) 38:712–6. 17075744

[10] Horner-Johnson W Biel FM Caughey AB Darney BG. Differences in prenatal care by presence and type of maternal disability. Am J Prev Med. (2019) 56:376–82. 10.1016/j.amepre.2018.10.021 30777157 PMC6402767

[11] Nishat F Lunsky Y Tarasoff LA Brown HK. Prenatal care adequacy among women with disabilities: a population-based study. Am J Prev Med. (2022) 62:39–49. 10.1016/j.amepre.2021.05.037 34426058 PMC9762461

[12] New global estimates on preterm birth published. WHO. Available online at: https://www.who.int/news/item/17-11-2018-new-global-estimates-on-preterm-birth-published (accessed November 17, 2018).

[13] WHO, March of Dimes, PMNCH, Save the Children. 15 million preterm births: Priorities for action based on national, regional and global estimates. In:Howson CP Kinney MV Lawn J, eds. Born Too Soon: The Global Action Report on Preterm Birth. (2012). Available online at: http://apps.who.int/iris/bitstream/handle/10665/44864/9789241503433_eng.pdfjsessionid=C96B31D83871F80D7E2023CF6A5A1495?sequence=1 (accessed March 9, 2021).

[14] Blencowe H Cousens S Oestergaard M Chou D Moller AB Narwal R. National, regional and worldwide estimates of preterm birth. Lancet. (2012) 379:2162–72. 10.1016/S0140-6736(12)60820-4 22682464

[15] Lumley J. Defining the problem: the epidemiology of preterm birth. BJOG. (2003) 110(Suppl 20):3–7. 12763104

[16] Annual Report on Birth Statistics 2018. Health Promotion Administration, Ministry of Health and Welfare (2019). Available online at: https://www.hpa.gov.tw/Pages/Detail.aspx?nodeid=649&pid=11780 (accessed March 9, 2021).

[17] Jiang M Mishu MM Lu D Yin X A. case control study of risk factors and neonatal outcomes of preterm birth. Taiwan J Obstet Gynecol. (2018) 57:814–8. 10.1016/j.tjog.2018.10.008 30545533

[18] Ruiz M Goldblatt P Morrison J Kukla L Svancara J Riitta-Järvelin M. Mother's education and the risk of preterm and small for gestational age birth: a DRIVERS meta-analysis of 12 European cohorts. J Epidemiol Community Health. (2015) 69:826–33. 10.1136/jech-2014-205387 25911693 PMC4552914

[19] Horner-Johnson W Kulkarni-Rajasekhara S Darney BG Dissanayake M Caughey AB. Live birth, miscarriage, and abortion among US women with and without disabilities. Disabil Health J. (2017) 10:382–6. 10.1016/j.dhjo.2017.02.006 28431989 PMC5544009

[20] Tarasoff LA Murtaza F Carty A Salaeva D Hamilton AD Brown HK. Health of newborns and infants born to women with disabilities: a meta-analysis. Pediatrics. (2020) 146:e20201635. 10.1542/peds.2020-1635 33203648 PMC7786829

[21] Teng SW Yen CF Liao HF Chang KH Chi WC Wang YH Taiwan I. C F T Evolution of system for disability assessment based on the international classification of functioning, disability, and health: a Taiwanese study. J Fla Med Assoc. (2013) 112:691–8. 10.1016/j.jfma.2013.09.007 24099681

[22] World Health Organization International classification of functioning, disability health: ICF. (2001). Available online at: https://apps.who.int/iris/bitstream/handle/10665/42407/9241545429.pdf (accessed December 23, 2020).

[23] Liu CY Hung YT Chuang YL Chen YJ Weng WS Liu JS. Incorporating development stratification of Taiwan townships into sampling design of large scale health interview survey. J Health Manag. (2006) 4:1–22. 10.29805/JHM.200606.0001

[24] Deyo RA Cherkin DC Ciol MA. Adapting a clinical comorbidity index for use with Icd-9-Cm administrative databases. J Clin Epidemiol. (1992) 45:613–9. 10.1016/0895-4356(92)90133-8 1607900

[25] Heazell AEP Newman L Lean SC Jones RL. Pregnancy outcome in mothers over the age of 35. Curr Opin Obstet Gynecol. (2018) 30:337–43. 10.1097/GCO.0000000000000494 30239372

[26] Lim NG Lee JY Park JO Lee J Oh J. Pregnancy, prenatal care, and delivery of mothers with disabilities in Korea. J Korean Med Sci. (2015) 30:127–32. 10.3346/jkms.2015.30.2.127 25653481 PMC4310936

[27] Redshaw M Malouf R Gao H Gray R. Women with disability: the experience of maternity care during pregnancy, labor, and birth and the postnatal period. BMC Pregnancy Childbirth. (2013) 13:174. 10.1186/1471-2393-13-174 24034425 PMC3848505

[28] Abele H Garnier Y Kuon R Maul H. Reducing the risk of preterm birth by ambulatory risk factor management. Dtsch Arztebl Int. (2019) 116:858–64. 10.3238/arztebl.2019.0858 31931955 PMC6970314

[29] Fairthorne J Bourke J O'Donnell M Wong K de Klerk N Llewellyn G. Pregnancy and birth outcomes of mothers with intellectual disability and their infants: advocacy needed to improve wellbeing. Disabil Health J. (2020) 13:100871. 10.1016/j.dhjo.2019.100871 31806497

[30] Höglund B Lindgren P Larsson M. Pregnancy and birth outcomes of women with intellectual disability in Sweden: a national register study. Acta Obstet Gynecol Scand. (2012) 91:1381–7. 10.1111/j.1600-0412.2012.01509.x 22881406 PMC3549474

[31] Mitra M McKee MM Akobirshoev I Valentine A Ritter G Zhang J. Pregnancy, birth, and infant outcomes among women who are deaf or hard of hearing. Am J Prev Med. (2020) 58:418–26. 10.1016/j.amepre.2019.10.012 31952943 PMC7039738

[32] Mueller BA Crane D Doody DR Stuart SN Schiff MA. Pregnancy course, infant outcomes, rehospitalization, and mortality among women with intellectual disability. Disabil Health J. (2019) 12:452–9. 10.1016/j.dhjo.2019.01.004 30692054 PMC6581578

[33] Quartermaine D Ed. Maternity Services for Women With Learning Difficulties. A Report of a Partnership of Midwives, Community Nurses and Parents. London: The Maternity Alliance (1999).

[34] Begley C Higgins A Lalor J Sheerin F. The Strengths and Weaknesses of Publicly-Funded Irish Health Services Provided to Women With Disabilities in Relation to Pregnancy, Childbirth, and Early Motherhood. Dublin: National Disability Authority (2010).

[35] Walsh-Gallagher D Sinclair M McConkey R. The ambiguity of disabled women's experiences of pregnancy, childbirth and motherhood: a phenomenological understanding. Midwifery. (2012) 28:156–62. 10.1016/j.midw.2011.01.003 21570753

[36] Clark KE Etomi O Ong VH. Systemic sclerosis in pregnancy. Obstet Med. (2020) 13:105–11. 10.1177/1753495X19878042 33093861 PMC7543163

[37] Vaught AJ Kovell LC Szymanski LM Mayer SA Seifert SM Vaidya D. Acute cardiac effects of severe pre-eclampsia. J Am Coll Cardiol. (2018) 72:1–11. 10.1016/j.jacc.2018.04.048 29957219 PMC8136241

[38] Lee H Okunev I Tranby E Monopoli M. Different levels of associations between medical comorbidities and preterm birth outcomes among racial/ethnic women enrolled in Medicaid 2014-2015: retrospective analysis. BMC Pregnancy Childbirth. (2020) 20:33. 10.1186/s12884-020-2722-8 31931778 PMC6958731

[39] Covarrubias LO Aguirre GER Chapuz JR May AIL Velázquez JD Eguiluz ME. Maternal factors associated to prematurity. Ginecol Obstet Mex. (2008) 76:526–36. 18798459

